# Mapping the landscape of research on insulin resistance: a visualization analysis of randomized clinical trials

**DOI:** 10.1186/s41043-024-00497-4

**Published:** 2024-01-09

**Authors:** Sa’ed H. Zyoud

**Affiliations:** 1 Department of Clinical and Community Pharmacy, College of Medicine and Health Sciences, An-Najah National University, Nablus, 44839 Palestine; 2 Clinical Research Centre, An-Najah National University Hospital, Nablus, 44839 Palestine

**Keywords:** Randomized clinical trials, RCTs, Insulin resistance, Insulin sensitivity, Bibliometric

## Abstract

**Background:**

Insulin resistance, a condition in which cells do not respond adequately to insulin, plays a crucial role in diabetes and related metabolic disorders. Randomized clinical trials (RCTs) explore interventions to manage insulin resistance, contributing to evidence-based medical progress. The current study aimed to analyze the global research landscape and trends in RCTs targeting insulin resistance.

**Methods:**

This study used bibliometric analysis and data visualization to examine RCT publications on insulin resistance from 2003 to 2022. The Scopus database was used due to its comprehensive coverage. The search strategy involved combining terms related to insulin resistance with RCT-related terms. The search query was validated, and core bibliometric indicators were used to analyze publication growth, origin, productivity, quality, and citations.

**Results:**

Between 2003 and 2022, 1077 RCT-focused publications on insulin resistance were identified from a pool of 24,932 related articles. The growth followed two phases, with a significant increase after 2008. The USA (*n* = 308; 28.60%), Iran (*n* = 165; 15.32%), China (*n* = 110; 10.21%), and the UK (*n* = 92; 8.54%) were the main contributors. The active institutions included *Tehran University of Medical Sciences* (*n* = 38; 3.53%) and *Harvard Medical School* (*n* = 31; 2.88%). Prominent funding agencies include the *National Institutes of Health* (*n* = 88; 8.17%) and the *National Institute of Diabetes and Digestive and Kidney Diseases* (*n* = 86; 7.99%). The top journals included the *American Journal of Clinical Nutrition* (*n* = 44; 4.09%) and *Diabetes Care* (*n* = 35; 3.25%). Co-occurrence analysis revealed three clusters addressing “utilizing lipid panels as indicators of insulin resistance,” “analyzing the impact of diet composition and physical activity on insulin sensitivity among obese individuals,” and “exploring insulin resistance in cases of polycystic ovary syndrome.”

**Conclusions:**

This comprehensive bibliometric analysis highlights the global research landscape and trends in RCTs targeting insulin resistance. Research on lipid panels, diet impact, and insulin resistance in patients with polycystic ovary syndrome will continue to be a hotspot. The findings offer valuable information on research priorities, international collaborations, and impactful publications. This study provides a foundation for future directorial investigations in this critical area of metabolic health.

## Background

Insulin resistance, also known as impaired insulin sensitivity, occurs when cells in the muscles, fat, or liver do not respond adequately to insulin [1]. The production of insulin by the pancreas is essential for the regulation of blood glucose levels and the sustenance of life [2]. The aforementioned ailment has the potential to be either transitory or chronic, and it can be effectively treated in specific instances [3]. Provided that the pancreas is capable of generating an adequate amount of insulin to counterbalance the diminishing cellular responsiveness, the levels of glucose in the bloodstream will be maintained within a state of optimal health. Nevertheless, if cells exhibit substantial resistance to insulin, it gives rise to heightened glucose levels in the bloodstream (hyperglycemia), ultimately culminating in the development of prediabetes and type 2 diabetes [4–6]. In addition, insulin resistance has been found to be correlated with several other illnesses, such as cardiovascular disease [7, 8], obesity [9, 10], nonalcoholic fatty liver disease [11, 12], polycystic ovarian syndrome [13, 14], and metabolic syndrome [15, 16].

Randomized clinical trials (RCTs) have traditionally served as the benchmark for collecting evidence in various medical domains because they objectively assess treatment effectiveness while minimizing the influence of external variables [17–21]. Ideally, every medical procedure or intervention should undergo rigorous evaluation through a robustly designed and adequately powered RCT. Nonetheless, there are instances where conducting RCTs faces obstacles, such as the need for timely evidence generation, financial constraints, limitations in study population diversity affecting applicability, ethical concerns, and the time required to complete these trials [21, 22]. Examining RCTs pertaining to insulin resistance offers a promising avenue for improving forthcoming approaches to addressing this issue. Bibliometric analysis and data visualization are commonly employed methods for objectively evaluating both quantitative and qualitative aspects of scientific research [23, 24].

Despite the extensive bibliometric analyses conducted on insulin resistance [25–29], there remains a notable gap in the investigation of RCTs on this subject. This study aimed to fill this void by examining the global landscape and trends surrounding insulin resistance RCTs. Through such an analysis, valuable insights can be gained into national research contributions, facilitating the comparison of research efforts and the identification of prevalent study patterns [23, 24]. Beyond enhancing academic understanding, bibliometric data can play a crucial role in informing resource allocation decisions among research organizations [23, 24].

The primary objective was to chart the global landscape of insulin resistance RCTs and pinpoint key trends. This scholarly resource provides researchers with data-driven insights into prominent trends, research gaps, and significant areas of concern. By offering a comprehensive understanding of the field, this study aims to inspire novel perspectives and guide future research endeavors.

The study aimed to explore the following research questions:

1. What domains or subject clusters are discernible in this field based on the terms utilized in publication titles and abstracts?
2. How has the research field evolved over time?
3. What are the primary research topics associated with publications on RCTs related to insulin resistance, and what connections exist between them?
4. What are the prominent journals, institutions, and countries in this field, and which ones are particularly prolific?
5. Which publications exert the greatest impact on this field?

## Methods

### Study design

Bibliometric methods were employed to perform a descriptive cross-sectional examination of publications on RCTs related to insulin resistance spanning the period from 2003 to 2022. Over the last decade, numerous scholarly publications have delved into bibliometric studies across diverse scientific domains [30–33]. Distinguishing itself from systematic reviews [34, 35], which target specific research queries using a restricted set of publications, and scoping reviews [36, 37], which aim to delineate the scope and nature of research evidence, bibliometric analysis serves as a valuable tool for capturing a momentary overview of both national and international contributions to the literature within a specific field. Moreover, this methodology provides essential data that assist in pinpointing gaps in research, thereby directing future studies toward potential areas of concentration [23, 38–40].

### Database used

For an optimal bibliometric investigation, it is crucial to employ various databases to analyze relevant documents comprehensively. However, when the volume of literature on the topic is extensive, it may not be feasible. A substantial volume of literature was found in the current study, necessitating the utilization of a single database. A literature review revealed that the Scopus database outperforms both PubMed and the Web of Science in terms of data size and the availability of functions for analysis and sorting [41–46].

Therefore, Scopus was utilized to achieve the research objectives. The advanced search function within Scopus was particularly advantageous, as it allowed us to construct long and intricate search queries. Generally, bibliometric studies rely on a single database due to the complexities of applying bibliometric indicators and mapping literature to documents retrieved from various databases. Specifically, Scopus provides complete coverage of PubMed and boasts double the number of indexed journals in comparison with Web of Science. As a result, Scopus is widely regarded as exceptionally comprehensive, encompassing numerous publications from both PubMed and Web of Science [44, 45].

### Search strategy

Relevant RCTs related to insulin resistance were identified through a thorough investigation in the Scopus database covering the period from January 1, 2003, to December 31, 2022. The last two decades were selected for analysis because they are presumed to offer a more comprehensive overview of publication and citation patterns. This timeframe facilitates the comparison of earlier and more recent periods, enabling the identification of shifts, growth, or declines in research output and impact. By confining the study to the years 2003–2022, researchers aimed to capture the latest trends in research on RCTs associated with insulin resistance. In addition, focusing on the most recent decades captures the latest advancements and emerging trends, ensuring that the study's findings are relevant and timely for researchers and practitioners. This period also covers a significant and crucial phase in research development within the field. Notably, research on RCTs involving patients with insulin resistance gained substantial attention and advancement after the year 2000 [47–49].

To minimize any potential biases due to database updates and changes, all relevant articles were retrieved on July 1, 2023. This study employed a precise and systematic search strategy incorporating advanced search techniques and a wide range of terms and phrases relevant to RCTs and insulin resistance.

**Fig. 1:**
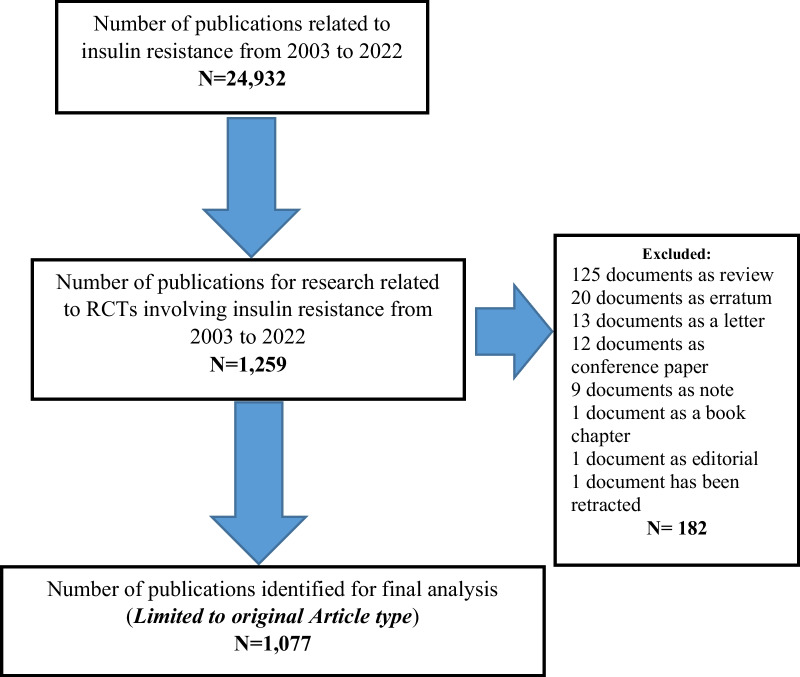
Flowchart for including and excluding literature studies

A flowchart depicting the specific selection procedures for the enrolled publications is presented in Fig. 1. The data for this study were recovered following the subsequent steps:

**Step 1**: Terms related to insulin resistance were selected from several previous systematic reviews and meta-analyses [50–52]; consequently, the terms "insulin resistance" and "insulin sensitivity" were included in the title of the article.

**Step 2**: The search results from the initial step were refined to encompass only documents containing the term 'randomized clinical trials' and associated terms within their titles or abstracts. The pertinent Medical Subject Headings (MeSH) for RCTs were acquired from PubMed's Medical Subject Headings (MeSH), in addition to prior systematic reviews and meta-analyses [53–58].

**Step 3**: The search focused exclusively on primary research articles, excluding other types of publications, such as errata, editorials, letters, and proceedings.

### Validation of the search query

Scientists have noted that including search terms in the title, rather than using a more extensive search covering title, abstract, and keywords, enhances specificity considerably with minimal impact on sensitivity [59–61]. A significant factor leading to the occurrence of false-positive results in keyword searches is the way Scopus treats keywords, including those identified as author keywords and indexed keywords such as "EMTREE drug terms," "EMTREE medical terms," and "Medline keywords." Therefore, the current study validated the search query using two criteria. First, two external colleagues in the field of bibliometric sciences judged the 100 most-cited documents and the documents with even numbers (110, 120, 130, 140, etc.) in the retrieved document list sent to them as an Endnote file (Endnote ™; version: 20, Clarivate Analytics, New York, NY, USA). The experts evaluated the presence of false-positive results, and in cases of disagreement, the principal investigator made the final judgment. The validity indicator was the absence of false-positive results, and the author refined the search query until both reviewers confirmed this absence.

The second validity criterion involved comparing the research output of the top twenty active authors with the number of documents relevant to RCTs on insulin resistance in their Scopus profiles. This comparison aimed to ensure the absence of false-negative results. The Pearson correlation test between the numbers and the actual numbers of the 20 selected authors showed a significant, positive, and strong correlation (*p* < 0.001, *r* = 0.965), indicating the high validity of the search strategy. Specifically, Sweileh et al. previously utilized this validation method [62–65].

## Bibliometric indicators

In this study, the core bibliometric indicators were classified into four main categories, which have been previously used in other published bibliometric studies [66–70]:

- *Overview of research output and growth of publications related to RCTs and insulin resistance:* This category encompassed the year of publication and the total citation count.
- *Origin and study patterns of publications:* This category focused on the productive countries contributing to research output.
- *Publication productivity:* Within this category, the study examined the leading institutions and funding agencies involved in this field.
- *Research impact and metrics:* This category evaluated the impact of frequently cited articles, the Hirsch index (h-index), and leading journals, along with their respective impact factors.

### Visualization analysis

VOSviewer software version 1.6.20, developed by Leiden University in the Netherlands, was utilized to generate network maps. These maps demonstrate the relationships between terms found in article titles or abstracts and collaborations between countries. VOSviewer was employed to construct knowledge networks based on solid scientific bases to predict upcoming research hotspots, revealing the progress of various research fields [71, 72]. By conducting co-occurrence analysis in VOSviewer, terms can be organized into distinct clusters, each represented by a unique color. As a result, performing cluster analysis on research hotspots becomes more effective through the co-occurrence network of terms in the title/abstract, allowing for identifying and illustrating emerging trends.

Apart from assessing the popularity of bibliometric items as a fundamental metric, we explore the links and both quantitative and qualitative research relationships among these items. Additionally, we utilized synthetic knowledge synthesis to generate concepts and themes, allowing us to identify hotspots in this field. Notably, Kokol and his colleagues have previously employed these pioneering methods [31, 73–76].

## Results

### Volume of publications

From 2003 to 2022, a total of 24,932 research articles on insulin resistance were published. However, when the search was narrowed down to articles specifically focused on RCTs related to insulin resistance, 1077 relevant publications were identified via Scopus (Fig. 1).

### Growth and productivity trends

**Fig. 2:**
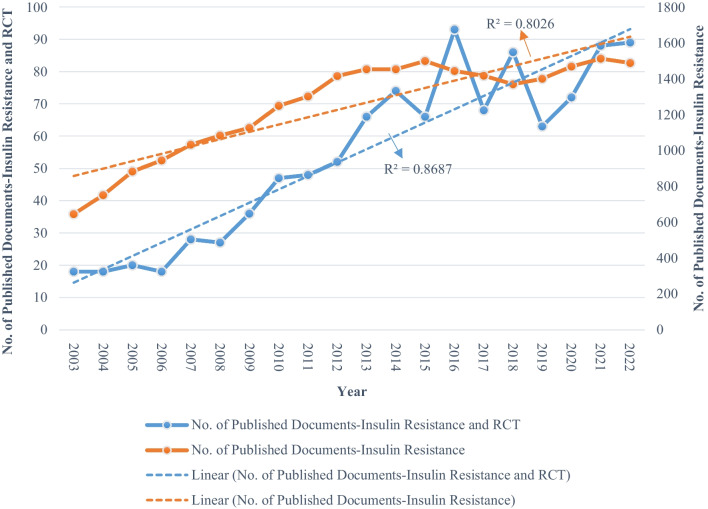
Trends in publications related to RCTs involving insulin resistance from 2003 to 2022

Figure 2 shows the research developments in RCTs focusing on insulin resistance in the past twenty years. The graph illustrates a notable increase in publications, starting at 18 in 2003 and reaching 93 in 2016. This growth occurred in two distinct phases: Initially, from 2003 to 2008, there was a relatively slow rate of publication, followed by a rapid increase from 2009 to 2022. Linear fit was obtained using simple linear regression (*R*^2^ = 0.869, *p* < 0.001), which revealed a modest positive correlation between the annual publication count and the corresponding year of publication.

### Performance of countries/regions

Table 1 displays a list of the top ten countries ranked by their research output. Upon closer inspection, it was discovered that out of 1077 publications distributed across 67 different countries. Notably, 308 publications were published in the USA, representing 28.60% of the total. Similarly, Iran occupies the second position with 165 publications (15.32%), followed by China with 110 publications (10.21%) and the UK with 92 publications (8.54%).

**Table 1:** The ten most active countries related to randomized clinical trials (RCTs) involving insulin resistance from 2003 to 2022

Ranking	Country	No. of documents	%^a^
1st	USA	308	28.60
2nd	Iran	165	15.32
3rd	China	110	10.21
4th	UK	92	8.54
5th	Canada	61	5.66
5th	Netherlands	61	5.66
7th	Italy	60	5.57
8th	Australia	47	4.36
9th	Mexico	45	4.18
10th	Germany	43	3.99

^a^There is overlapping research productivity among countries, which occurs when researchers from different countries collaborate on research projects or when the same research output is credited to multiple countries

**Fig. 3:**
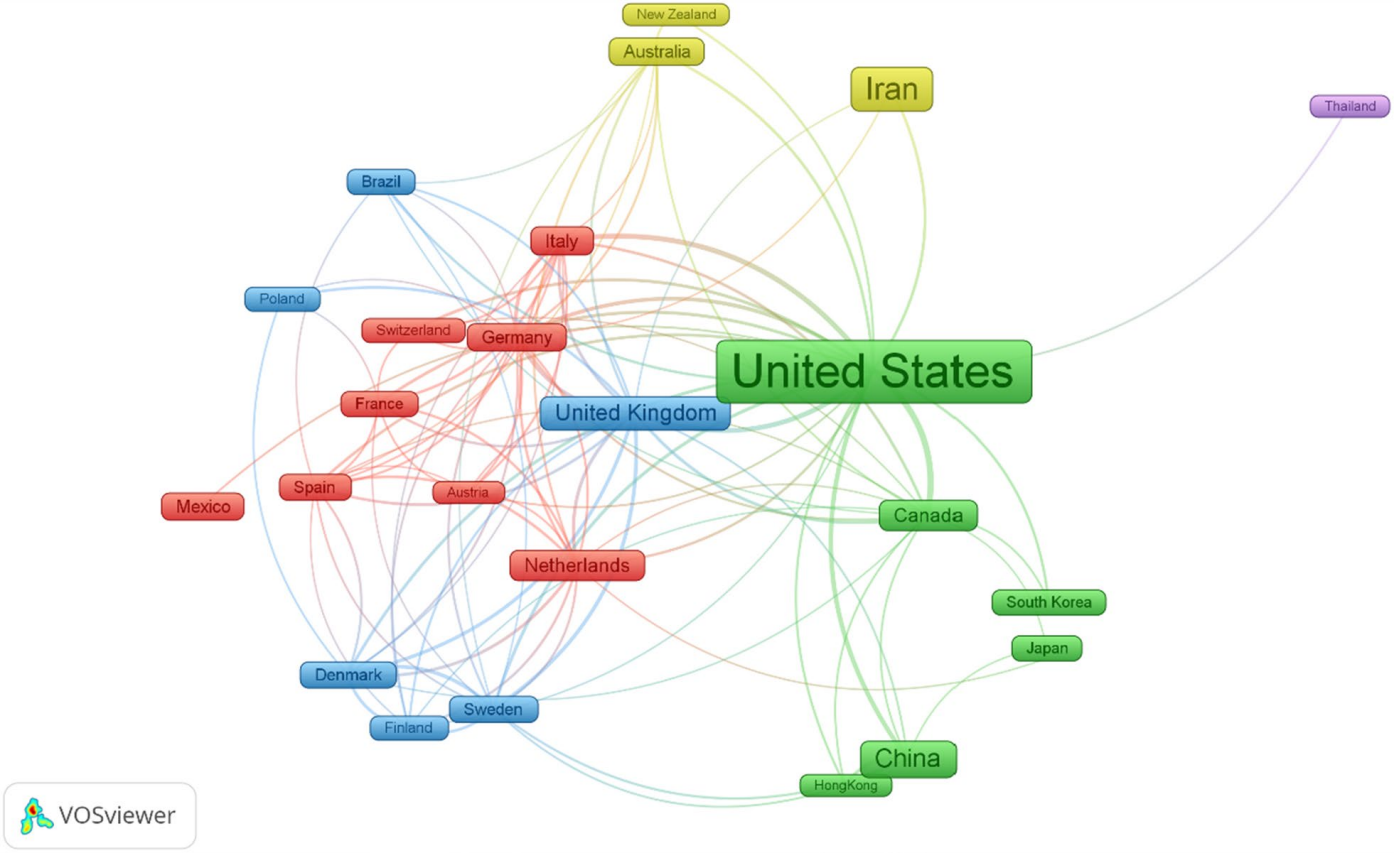
International (cross-country) research collaboration between countries with a minimum contribution of 10 articles. There were 25 countries on the map

Figure 3 demonstrates the extent of global research cooperation among countries, highlighting that a minimum of 10 articles contributed to the research domain. The map encompasses 25 different countries, and centrally positioned countries such as the USA and China exhibit larger nodes, signifying a greater number of documents involving international collaborative efforts.

### Active institutions/organizations

Table 2 shows the leading ten institutions out of 4,230 that actively contributed to research publications on RCTs and insulin resistance. Taken together, these institutions were responsible for 17.18% of all the documents published in this field. *Tehran University of Medical Sciences* was the first to produce 38 documents, representing 3.53% of the total publications. Similarly, *Harvard Medical School* had 31 publications (2.88%), while *Tabriz University of Medical Sciences* and *Brigham and Women's Hospital* both produced 27 publications (2.51%) and 25 publications (2.32%), respectively. The majority of the active institutions were from Iran, represented by four institutions, followed by two institutions from the USA and two from Mexico and Denmark, represented by one institution.

**Table 2:** The top ten active institutions in related randomized clinical trials (RCTs) involving insulin resistance from 2003 to 2022

Ranking	Institute	Country	No. of documents	%^a^
1st	Tehran University of Medical Sciences	Iran	38	3.53
2nd	Harvard Medical School	USA	31	2.88
3rd	Tabriz University of Medical Sciences	Iran	27	2.51
4th	Brigham and Women's Hospital	USA	25	2.32
5th	Københavns Universitet	Denmark	23	2.14
6th	Instituto Mexicano del Seguro Social	Mexico	22	2.04
6th	Universidad de Guadalajara	Mexico	22	2.04
6th	Shahid Beheshti University of Medical Sciences	Iran	22	2.04
6th	Isfahan University of Medical Sciences	Iran	22	2.04
6th	Nutrition Research Center	Iran	22	2.04

^a^There is overlapping research productivity among institutions, which occurs when researchers from different institutions collaborate on research projects or when the same research output is credited to multiple institutes

### Top funding agencies

Table 3 shows the ten leading funding agencies that significantly contributed to this field. In particular, the *National Institutes of Health* (*n* = 88; 8.17%), the N*ational Institute of Diabetes and Digestive and Kidney Diseases* (*n* = 86; 7.99%), and the *National Center for Research Resources* (*n* = 63; 5.85%) have emerged as major contributors in terms of funding. It should be noted that the USA has played a major role in this field, as most active funding agencies are based in the country, with seven of the represented agencies being from the USA.

**Table 3:** The top ten funding agencies that published the most randomized clinical trials (RCTs) involving insulin resistance between 2003 and 2022

Ranking	Funding agencies	Country	No. of documents	%^a^
1st	National Institutes of Health	USA	88	8.17
2nd	National Institute of Diabetes and Digestive and Kidney Diseases	USA	86	7.99
3rd	National Center for Research Resources	USA	63	5.85
4th	National Heart, Lung, and Blood Institute	USA	45	4.18
5th	National Center for Advancing Translational Sciences	USA	38	3.53
6th	National Institute on Aging	USA	32	2.97
7th	National Natural Science Foundation of China	China	26	2.41
8th	Medical Research Council	UK	22	2.04
9th	Eunice Kennedy Shriver National Institute of Child Health and Human Development	USA	21	1.95
10th	National Health and Medical Research Council	Australia	15	1.39
10th	Novo Nordisk	Denmark	15	1.39

^a^There is overlapping research productivity among funding agencies, which happens when researchers from different funding agencies collaborate on research projects or when the same research output is attributed to multiple funding agencies

### Top active journals

Table 4 illustrates the leading ten journals, arranged according to their publication count. These journals contributed 291 articles, representing approximately 27.02% of all the publications. The *American Journal of Clinical Nutrition* holds the top position, publishing 44 articles, representing 4.09% of the total. There were 35 articles on *Diabetes Care* (3.25%), 32 on the *Journal of Clinical Endocrinology and Metabolism* (2.97%), and 26 on the *Diabetologia* (2.41%).

**Table 4:** The leading journals with the most publications on randomized clinical trials (RCTs) involving insulin resistance from 2003 to 2022

Ranking	Journal/source title	No. of documents	%	IF^*^
1st	American Journal of Clinical Nutrition	44	4.09	7.1
2nd	Diabetes Care	35	3.25	16.2
3rd	Journal of Clinical Endocrinology and Metabolism	32	2.97	5.8
4th	Diabetologia	26	2.41	8.2
5th	Diabetes Obesity and Metabolism	24	2.23	5.8
6th	British Journal of Nutrition	23	2.14	3.6
7th	Clinical Nutrition	21	1.95	6.3
7 h	Nutrients	21	1.95	5.9
9th	Plos One	20	1.86	3.7
10th	Diabetes	15	1.39	7.7
10th	Journal of Nutrition	15	1.39	4.2
10th	Trials	15	1.39	2.5

*2022 Journal Citation Reports™ (Clarivate, 2023)

### Analysis of citations

Based on the citation analysis, the articles that were retrieved garnered an average of 43.87 citations per article. This led to an h-index of 105 and a cumulative total of 47,251 citations. Among these articles, 60 did not receive any citations, while 113 received more than 100 citations. The citation counts for these articles varied from 0 to 2027. Table 5 shows the top ten RCTs and insulin resistance publications, which together had 6,894 citations. The citation range for these publications is 479 to 1977 [77–86].

**Table 5:** The ten most-cited articles that cited randomized clinical trials (RCTs) on insulin resistance between 2003 and 2022

Authors	Title	Year	Source title	Cited by
Vrieze et al. [86]	“Transfer of intestinal microbiota from lean donors increases insulin sensitivity in individuals with metabolic syndrome”	2012	Gastroenterology	2027
Sutton et al. [84]	“Early Time-Restricted Feeding Improves Insulin Sensitivity, Blood Pressure, and Oxidative Stress Even without Weight Loss in Men with Prediabetes”	2018	Cell Metabolism	666
Kapoor et al. [80]	“Testosterone replacement therapy improves insulin resistance, glycemic control, visceral adiposity and hypercholesterolaemia in hypogonadal men with type 2 diabetes”	2006	European Journal of Endocrinology	642
Merovci et al. [81]	“Dapagliflozin improves muscle insulin sensitivity but enhances endogenous glucose production”	2014	Journal of Clinical Investigation	595
Brasnyó et al. [78]	“Resveratrol improves insulin sensitivity, reduces oxidative stress and activates the Akt pathway in type 2 diabetic patients”	2011	British Journal of Nutrition	511
Von Hurst et al. [85]	“Vitamin D supplementation reduces insulin resistance in South Asian women living in New Zealand who are insulin resistant and vitamin D deficient-a randomized, placebo-controlled trial”	2010	British Journal of Nutrition	500
Ross et al. [82]	“Exercise-induced reduction in obesity and insulin resistance in women: A randomized controlled trial”	2004	Obesity Research	494
Grassi et al. [79]	“Cocoa reduces blood pressure and insulin resistance and improves endothelium-dependent vasodilation in hypertensives”	2005	Hypertension	495
Baker et al. [77]	“Insulin resistance and Alzheimer-like reductions in regional cerebral glucose metabolism for cognitively normal adults with prediabetes or early type 2 diabetes”	2011	Archives of Neurology	485
Ryan et al. [83]	“The Mediterranean diet improves hepatic steatosis and insulin sensitivity in individuals with nonalcoholic fatty liver disease”	2013	Journal of Hepatology	479

### Co-occurrence analysis

**Fig. 4:**
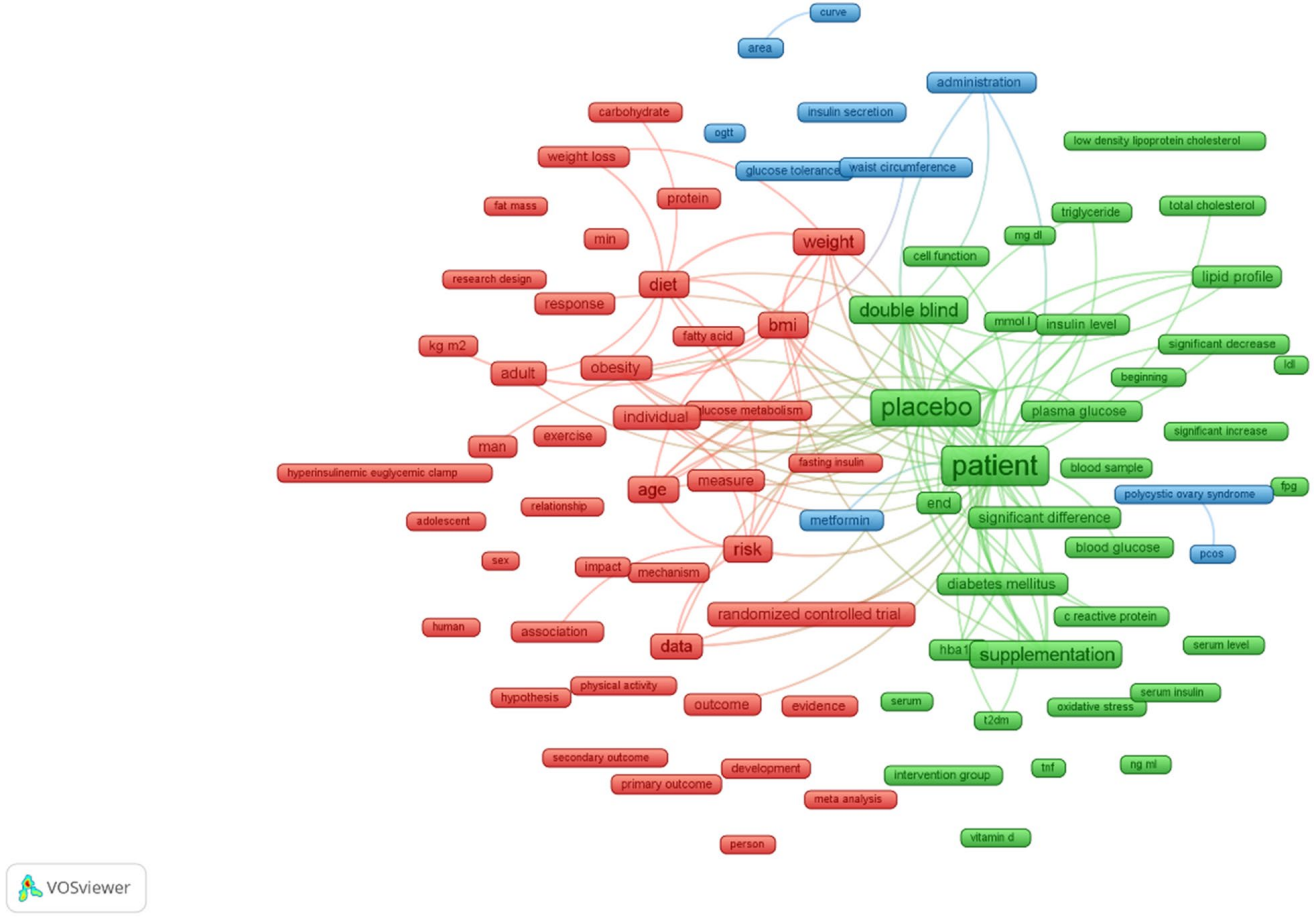
Visualization of the research themes: VOSviewer 1.6.20-generated map illustrating diverse clusters extracted from the title and abstract term analysis. Clustering diversity revealed through color variation and term frequency displayed by circle size

VOSviewer was used to conduct co-occurrence analysis of the title and abstract content of publications, specifically focusing on RCTs related to insulin resistance. The visualizing research themes are shown in Fig. 4, and the synthesis of the results is shown in Table 6. Several research topics in publications focusing on RCTs related to insulin resistance have been presented via network visualization by mapping more than 40 times the co-occurrence of terms in the title/abstract in Scopus database publications. A total of 172 out of the 20,596 terms reached the threshold, and this set of terms was scattered into three different clusters (Fig. 4). Each cluster is represented by different colors (green for Cluster 1, red for Cluster 2, and blue for Cluster 3) and encompasses terms related to specific research topics. Cluster 1 focused on "lipid panels as a marker of insulin resistance," Cluster 2 focused on the "effect of diet and exercise composition on insulin sensitivity in obese subjects," and Cluster 3 addressed "insulin resistance in polycystic ovary syndrome (PCOS).” In particular, the terms within each cluster exhibited strong connections, highlighting the coherence of the research within these themes.

**Table 6:**
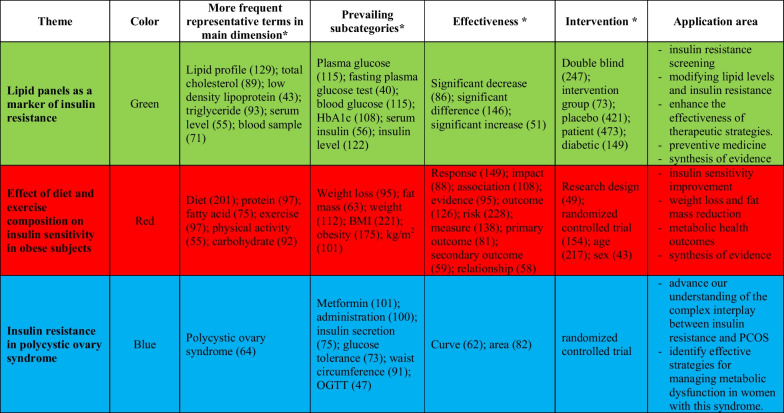
Analyzing cooccurring terms in titles and abstracts of publications: a synthetic knowledge synthesis for concept and theme generation in randomized clinical trials for insulin resistance (2003–2022)

*Numbers in parentheses represent the number of papers in which the terms were obtained from the title and abstract

### Future research direction analysis

**Fig. 5:**
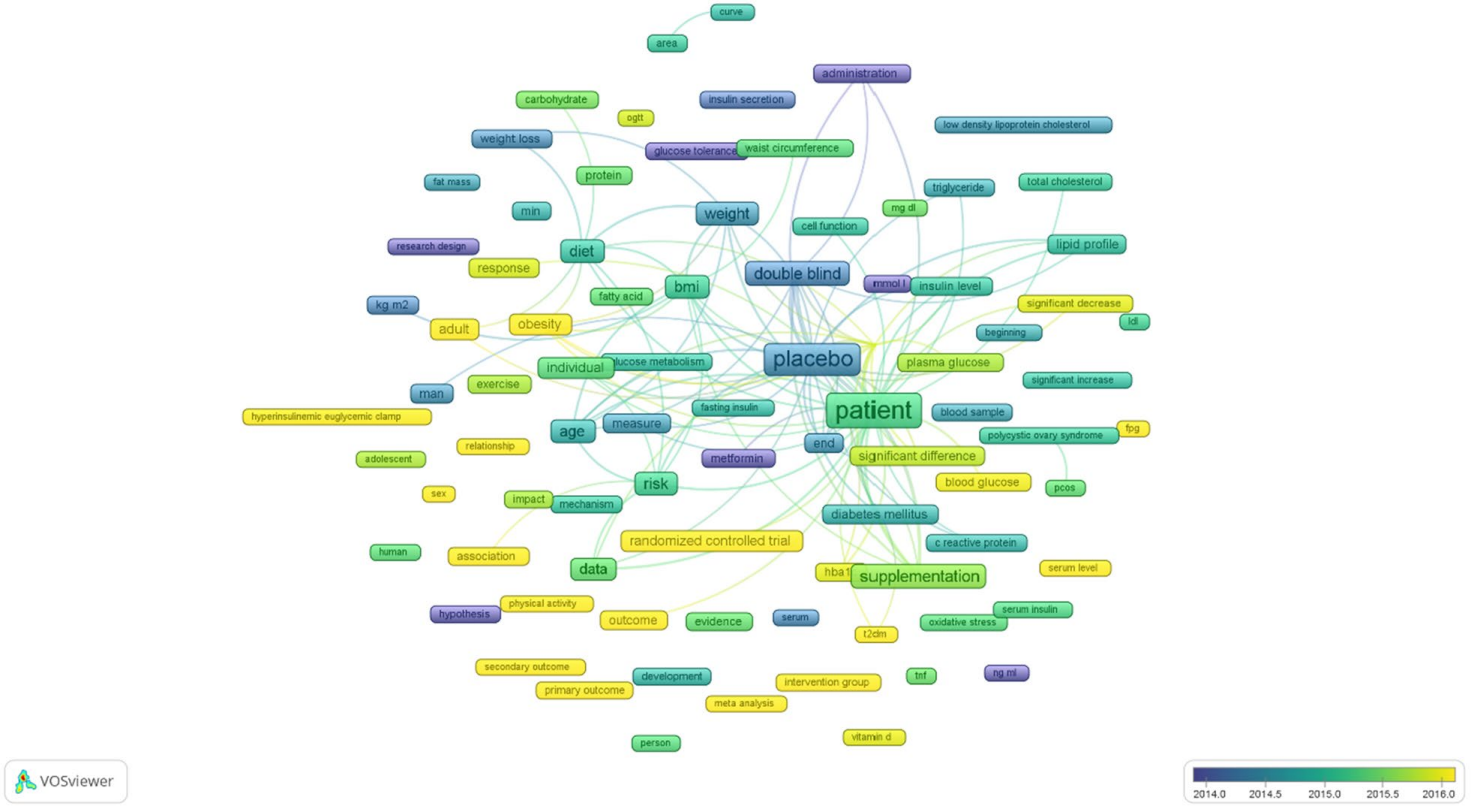
Mapping term co-occurrence over time: a visual representation of term frequency in titles and abstracts using VOSviewer 1.6.20. The blue nodes represent earlier occurrences, and the yellow nodes represent later occurrences

The terms employed in research publications focused on RCTs related to the progression of insulin resistance over time were also identified within the titles and abstracts in the investigation of term co-occurrence (Fig. 5). In particular, studies exploring the impact of diet composition and physical activity on insulin sensitivity in obese individuals, as well as the use of lipid panels as an indicator of insulin resistance, have emerged in more recent years (post-2015) than earlier publications focused on insulin resistance in patients with polycystic ovary syndrome (pre-2015).

## Discussion

This study is the first to use bibliometric analysis to systematically investigate the characteristics of research publications on RCTs related to insulin resistance over a two-decade period (2003–2022). The study also identified the top research priorities in this field. This knowledge will help guide future research on insulin resistance.

In the field of insulin resistance, there has been an explosion of scientific articles focused on CRTs. This increase can be attributed to the growing international interest and to the influx of researchers from various backgrounds. This review provides a comprehensive analysis of the current RCT landscape investigating insulin resistance, allowing scholars to identify promising research avenues and develop effective solutions to this urgent public health issue. The review revealed a significant geographical disjunction in RCT output, with concentrations in certain countries and regions. The USA, China, and the UK are likely to dominate the field due to their robust research infrastructure and significant scientific investment. These countries have a critical mass of well-funded and competent researchers, contributing to their leading position in research publications on RCTs related to insulin resistance [87, 88].

Interestingly, the analysis differs from traditional metrics by revealing that Iran is a remarkable contributor to RCT research into therapeutic interventions for insulin resistance in developing countries [89, 90]. This success can be attributed to several factors: (1) A targeted research funding initiative for medications to treat insulin resistance may have played an important role; (2) Iran's famous universities and medical institutes produce trained researchers and health professionals capable of administering high-quality drugs; and (3) Iranian health authorities recognize the importance of insulin resistance in public health and may have prioritized drugs that address this problem [91–93]. The global momentum toward insulin-resistant TCRs opens up exciting possibilities for future research and the development of effective interventions. Understanding the various factors that affect research results across countries, including Iran's unique contributions, can inform the development of effective strategies to promote this area and address this vital public health challenge.

Moreover, the increase in the number of research articles focusing on RCTs concerning insulin resistance can be attributed to the reality that multiple trending subjects were published in the same timeframe [77–86]. These topics unveiled new and innovative hypotheses and laid the foundation for emerging areas of study, including concepts such as "utilizing lipid panels as indicators of insulin resistance", "analyzing the impact of diet composition and physical activity on insulin sensitivity among obese individuals” and "exploring insulin resistance in cases of polycystic ovary syndrome."

The use of lipid panels as indicators of insulin resistance has emerged as a prominent topic in publications related to RCTs investigating insulin resistance. The present approach recognizes that dyslipidemia, characterized by lipid level deviations, frequently coexists with insulin resistance. Several RCTs have investigated the association between lipid profiles and insulin resistance [94–97]. Insulin resistance is commonly associated with increased triglyceride and low-density lipoprotein cholesterol (LDL-C) levels. However, a strong association exists between elevated high-density lipoprotein cholesterol (HDL-C) levels and increased insulin sensitivity [17, 98–100].

The influence of diet and physical activity composition on insulin sensitivity in obese individuals is a prominent topic in the scholarly literature on RCTs focused on insulin resistance. Several studies have shown that low-carbohydrate diets can result in increased insulin sensitivity in individuals who are obese [101–106]. Limitation of carbohydrate intake has been shown to potentially contribute to regulating blood glucose levels and mitigating insulin resistance [107, 108]. Evidence suggests that diets high in fiber, specifically those that contain large amounts of whole grains, vegetables, and legumes, are associated with increased insulin sensitivity [109, 110]. Dietary fiber plays a crucial role in moderating the sugar absorption rate, thus mitigating abrupt increases in blood glucose levels [111]. Several studies have shown that caloric restriction and subsequent weight loss can effectively enhance insulin sensitivity in obese individuals [112–115]. The attainment of a healthy body weight can significantly influence the management of insulin resistance.

Insulin resistance in individuals diagnosed with PCOS has garnered considerable attention in the context of RCTs and the associated literature. The research theme underscores the importance of comprehending the correlation between insulin resistance and PCOS, as it has implications for managing and treating this prevalent endocrine disorder in women. The phenomenon of insulin resistance in PCOS has been extensively documented in the literature [116, 117]. PCOS is a multifaceted endocrine disorder frequently characterized by insulin resistance, resulting in elevated insulin concentrations within the circulatory system. These factors can potentially contribute to various metabolic and reproductive complications, such as obesity, disrupted menstrual cycles, and infertility [118, 119].

### Clinical perspectives and implications for the future

The current study examined a range of controversial subjects related to insulin resistance through a visual analysis of RCTs to clarify the existing knowledge and suggest possible directions for future investigations. Its clinical application comprises several critical domains (Table 6). The primary objective of most studies in this field is to investigate various approaches for detecting insulin resistance, with a particular focus on identifying diagnostic tools or techniques that can precisely identify patients afflicted with insulin resistance. Furthermore, the research investigates methodologies or approaches intended to alter lipid levels with the purpose of mitigating insulin resistance. Additionally, these studies investigated strategies to improve the efficacy of therapeutic interventions targeting insulin resistance, which may entail the development of innovative treatments or the refinement of preexisting treatments. Exploring preventive strategies and techniques to reduce the risk of insulin resistance is crucial. This encompasses various approaches, such as lifestyle modifications, medication administration, and other preventive methodologies. Furthermore, specific studies have explored the connection between insulin resistance and PCOS, with the goal of improving the understanding of the intricate interplay between these two conditions.

### Limitations

The current study is the first to present baseline data on research activity on the global research landscape and trends concerning RCTs focused on insulin resistance. This study has several limitations [120, 121]. Discrepancies among databases, differences within disciplines, and an English-language bias represent intrinsic constraints in bibliometric analysis [122, 123]. Limiting the search to titles containing the phrase "insulin resistance or insulin sensitivity" may have led to the oversight of related publications utilizing these concepts as keywords, similar to previous research [29, 124]. The use of different databases, such as Google Scholar, Scopus, PubMed, and Web of Science, to record varying numbers of publications further complicates such studies. Additionally, focusing solely on the quantity of articles neglects quality elements such as relevance, impact, and reliability. Analyzing overall citation numbers without considering yearly averages risks missing recent high-quality research that has not yet impacted the field. Moreover, the preference for English as the language of science raises the possibility of excluding articles in other languages, particularly in regions such as the Eastern Mediterranean (EMRO), African Region (AFRO), and South‒East Asia Region (SEARO), where Scopus underrepresents regional journals. Finally, variations in institution names or forms may lead to uneven research output, potentially excluding institutions from the "prolific list." Recognizing these limitations is vital for understanding their potential impact on the precision and inclusiveness of current results. Consequently, these limitations do not significantly compromise the overall validity of the study.

## Conclusions

This comprehensive bibliometric analysis provides valuable insights into the global research landscape and trends concerning RCTs focused on insulin resistance. This study demonstrated the substantial growth of related research in this field over the last two decades, during which a notable surge in publication activity occurred after 2008. Key contributors to this research include the USA, Iran, China, and the UK, with institutions such as *Tehran University of Medical Sciences* and *Harvard Medical School*, along with funding agencies such as the *National Institutes of Health*, which play pivotal roles. By utilizing co-occurrence analysis, three prominent research clusters were identified, emphasizing the significance of exploring lipid panels as indicators of insulin resistance, assessing the impact of diet composition and physical activity on insulin sensitivity in obese individuals, and investigating insulin resistance in patients with PCOS. These clusters represent the current and emerging research priorities in the field. This study sheds light on research patterns and collaborations and emphasizes the significance of international cooperation and interdisciplinary investigation. The findings are useful for researchers, policymakers, and healthcare professionals interested in metabolic health. As insulin resistance research continues to advance, fueled by the knowledge gained from RCTs, advances in preventing, diagnosing, and treating metabolic disorders are expected. This study serves as a foundational resource, guiding future research and promoting the use of evidence-based methods to address the complex world of insulin resistance.

## Data Availability

All the data generated or analyzed during this study are included in this published article. In addition, other datasets used during the current study are available from the author upon reasonable request (saedzyoud@yahoo.com).

## Abbreviations

RCTs: Randomized clinical trials
PCOS: Polycystic ovary syndrome
LDL-C: Low-density lipoprotein cholesterol
HDL-C: High-density lipoprotein cholesterol
MeSH: Medical Subject Headings
EMRO: Eastern Mediterranean Region
AFRO: African Region
SEARO: South‒East Asia Region
EMTREE: Embase's Thesaurus
